# Sepsis-induced acute kidney injury by standardized colon ascendens stent peritonitis in rats - a simple, reproducible animal model

**DOI:** 10.1186/s40635-014-0034-x

**Published:** 2014-12-09

**Authors:** Martin A Schick, Wolfgang Baar, Sven Flemming, Nicolas Schlegel, Jakob Wollborn, Christopher Held, Reinhard Schneider, Robert W Brock, Norbert Roewer, Christian Wunder

**Affiliations:** Department of Anaesthesia and Critical Care, University of Würzburg, Oberdürrbacherstraße 6, 97080 Würzburg, Germany; Department for Anesthesiology and Critical Care Medicine, University of Freiburg, Hugstetter Straße 55, 79106 Freiburg, Germany; Department of General, Visceral, Vascular and Paediatric Surgery (Department of Surgery I), University of Würzburg, Oberdürrbacherstraße 6, 97080 Würzburg, Germany; Department of Internal Medicine I, Division of Nephrology, University Hospital Würzburg, Oberdürrbacherstraße 6, 97080 Würzburg, Germany; Department of Physiology and Pharmacology, West Virginia University School of Medicine, Robert C. Byrd Health Science Center, P.O. Box 9105, 26506 Morgantown, WV USA

**Keywords:** Sepsis, Acute kidney injury, Animal model, CASP

## Abstract

**Background:**

Up to 50% of septic patients develop acute kidney injury (AKI). The pathomechanism of septic AKI is poorly understood. Therefore, we established an innovative rodent model to characterize sepsis-induced AKI by standardized colon ascendens stent peritonitis (sCASP). The model has a standardized focus of infection, an intensive care set up with monitoring of haemodynamics and oxygenation resulting in predictable impairment of renal function, AKI parameters as well as histopathology scoring.

**Methods:**

Anaesthetized rats underwent the sCASP procedure, whereas sham animals were sham operated and control animals were just monitored invasively. Haemodynamic variables and blood gases were continuously measured. After 24 h, animals were reanesthetized; cardiac output (CO), inulin and PAH clearances were measured and later on kidneys were harvested; and creatinine, urea, cystatin C and neutrophil gelatinase-associated lipocalin (NGAL) were analysed. Additional sCASP-treated animals were investigated after 3 and 9 days.

**Results:**

All sCASP-treated animals survived, whilst ubiquitous peritonitis and significantly deteriorated clinical and macrohaemodynamic sepsis signs after 24 h (MAP, CO, heart rate) were obvious. Blood analyses showed increased lactate and IL-6 levels as well as leucopenia. Urine output, inulin and PAH clearance were significantly decreased in sCASP compared to sham and control. Additionally, significant increase in cystatin C and NGAL was detected. Standard parameters like serum creatinine and urea were elevated and sCASP-induced sepsis increased significantly in a time-dependent manner. The renal histopathological score of sCASP-treated animals deteriorated after 3 and 9 days.

**Conclusions:**

The presented sCASP method is a standardized, reliable and reproducible method to induce septic AKI. The intensive care set up, continuous macrohaemodynamic and gas exchange monitoring, low mortality rate as well as the opportunity of detailed analyses of kidney function and impairments are advantages of this setup. Thus, our described method may serve as a new standard for experimental investigations of septic AKI.

## Background

Acute kidney injury (AKI) is common and imposes a heavy burden of illness (morbidity and mortality) [1]. Thirty to fifty percent of septic patients develop renal failure, and AKI can double the mortality rate up to 70%. Only a few proven therapeutic interventions and preventions of AKI exist, and survivors with end-stage renal impairment suffer from reduced quality of life [2]. Additional health care cost for example dialysis, transplantation and further intensive care admissions are enormous. The pathophysiology of septic AKI is poorly understood. On the one hand, AKI can occur due to macrohaemodynamic changes with hypotension, reduced renal blood flow (RBF) following global or regional renal ischemia. On the other hand, there is evidence that septic AKI can develop without clinical signs of septic shock [3]. Standardized animal models are necessary to gain insights into the pathogenic renal mechanisms associated with sepsis. An ideal AKI animal sepsis model should base upon (a) standardized focus of infection as a septic origin in a clinical setting [4], (b) invasive monitoring of macrohaemodynamics, (c) investigation of RBF with following renal function tests, (d) histopathology and (e) feasible reproducibility.

Therefore, we conducted this study to develop a new rodent preparation in which all aspects mentioned above are implemented to characterize sepsis-induced AKI by standardized colon ascendens stent peritonitis (sCASP).

## Methods

### Animals

After the Animal Care Committee approval (Laboratory Animal Care and Use Committee of the District of Unterfranken, Germany), experiments were performed on 30 male Sprague-Dawley rats (308 ± 21 g bodyweight (BW), 8 to 12 weeks old) purchased from Harlan Winkelmann (Borchen, Germany). All rats were maintained on a standard diet, water *ad libitum*, 12-h day and night cycles and were not fasted prior to the experiment. Animals were randomized to groups I to III (group I: control, *n* = 6; group II: sham, *n* = 6; group III: sCASP, *n* = 14), anaesthetized and prepared as follows.

### Exclusion criteria

Animals which died or exceeded severe stress before the end of the experiment or exhibited MAP levels <60 mmHg during the 24 h of observation were excluded from the study (*n* = 3). The reasons for the fulfilment of these exclusion criteria were bleedings during the surgical procedures.

### Invasive monitoring and continuous medication

*Day 0:* Rats were anaesthetized using isoflurane (Forene®, Abbott, Germany)/nitrous oxide inhalation. To administer fluids and drugs, the right jugular vein was cannulated as the left carotid artery was used for continuous measurement of the blood pressure and the heart rate (Hewlett Packard Model 88S; Hewlett-Packard, Palo Alto, CA, USA) as well as to gain blood samples. Afterwards, the catheters were tunnelled subcutaneously to the neck and connected to Swivel tethers (NeuroLab, St. Louis, MO, USA). After surgery, all animals received intravenous analgesia using Fentanyl (group I-II: 0.25 μg/100 g BW/h fentanyl (Fagron, Barsbuettel, Germany); group III: 2.0 μg/100 g BW/h), and all animals received 14.4 ml/kg BW/24 h NaCl 0.9% (group I-III; Fresenius Kabi, Germany) for basal fluid requirement until tracheotomy and ventilation. After surgery, animals were awake and able to move freely in the cage, whilst being invasively monitored and continuously medicated with free access to water *ad libitum*.

**Figure 1:**
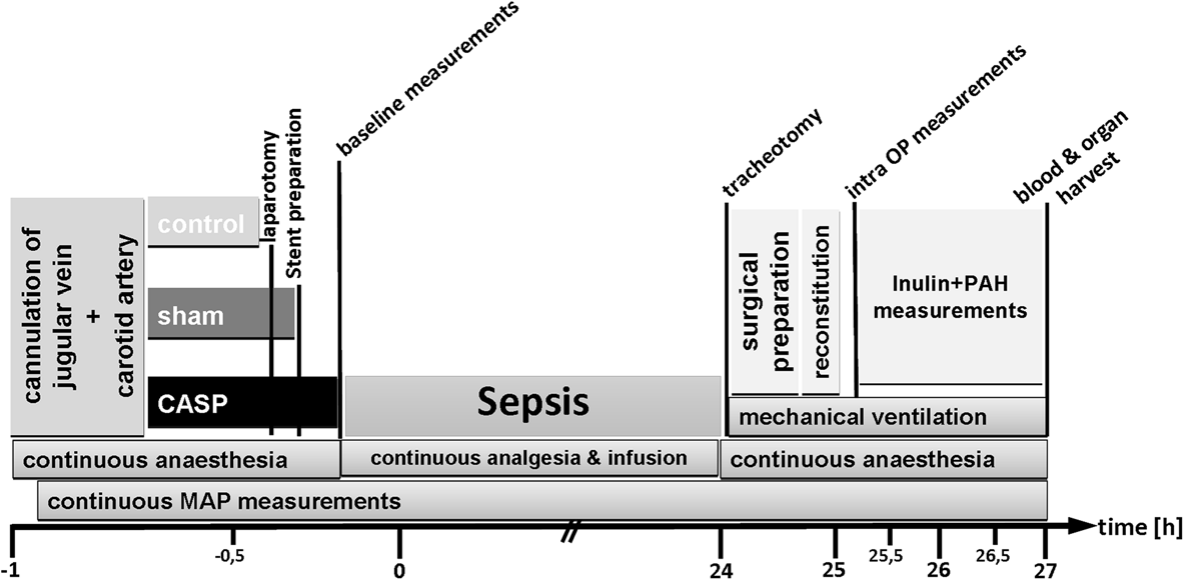
**Timetable of study setting.**

*Day 1:* After 24 h (timetable see Figure 1), rats were reanaesthetized using Midazolam (Midazolam-ratiopharm®, (Ratiopharm, Ulm, Germany) 0.7 mg/100 g BW/h) and Fentanyl (7 μg/100 g BW/h). For sufficient oxygenation and ventilation (SaO_2_ > 93%, PaO_2_ > 60 mmHg, PaCO_2_ 35 to 45 mmHg), a tracheotomy was performed, and the rats were mechanically ventilated with FiO_2_ 0.28 using a Rodent Ventilator (type: 7025, Hugo Sachs Eletronik KG, March-Hugstetten, Germany). For measurements of the cardiac output (CO), the right femoral artery was cannulated and a thermocatheter (MLT1402 T-type Ultra Fast Thermocouple, ADInstruments, Oxford, UK) was introduced. CO was measured by thermodilution using PowerLab® (ADInstruments Ltd, Oxford, United Kingdom). Parameters were calculated as described previously [5,6]. To measure renal blood flow (RBF), the left renal artery was separated from the renal vein and an ultrasonic flow probe (MA1PRB, Transonic Systems, Ithaca, NY, USA) was installed. Blood gas values were measured using ABL505 blood gas analyser (Radiometer, Copenhagen, Denmark).

### Standardized colon ascendens stent peritonitis and sham procedure

**Figure 2:**
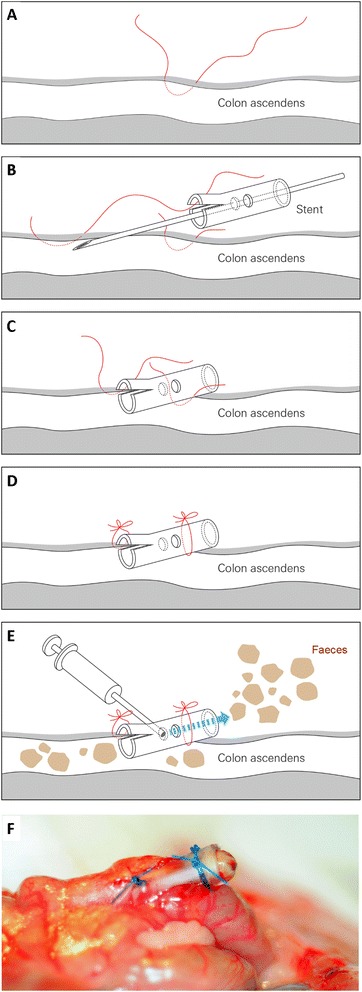
**Insertion of the stent into the ascending colon.** Prepare the fixation suture **(A)** before starting the insertion of the stent into the ascending colon **(B)**. Having reached the final position fix, the stent with the prepared sutures **(C)** and **(D)**. Make sure to get a systemic inflammation by distributing the faeces using 2 ml of 0.9% sodium chloride **(E)**. **(F)** shows the final position of the stent during one of our surgeries.

On day 0, animals of group III received the sCASP procedure (timetable see Figure 1). Anaesthetized rats, with cannulated carotid artery and jugular vein, were placed in supine position, and a midline abdominal incision was performed to open the peritoneal cavern along the linea alba. After identification of the caecal pole, the caecum was gently pulled out. Two-centimetre distal from the ileocaecal valve, the wall of the ascending colon was pierced with a suture at the anti-mesenteric side (Figure 2). The colon was punctured with a 14G needle, and to ensure the occurrence of a continuous faecal peritonitis, a 10 FR plastic tube (tip of a suction catheter, type ‘Ideal’, B.Braun Melsungen, Melsungen, Germany) was inserted. Stool was milked from the caecum towards the colonic stent until it appeared at the outlet of the stent. The stent was flushed with 2-ml NaCl 0.9% (Fresenius Kabi, Bad Homburg, Germany) to distribute the faeces into peritoneal cavern. Animals of group II were sham operated. The peritoneal cavity was opened, but in contrast to the sCASP procedure, the colon remained closed and the stent was only fixed at the outside of the colonic wall.

### Evaluation of kidney function *in vivo*

Inulin and PAH clearances were determined as described recently [7]. In brief, fluorescein-isothiocyanate-inulin (Inulin-FITC; F3272-1; Sigma-Aldrich; St. Louis, MO, USA) and PAH (p-Aminohippuric acid sodium salt; A3759-25G; Sigma-Aldrich; St. Louis, MO, USA) (1 mg of each substance solved in 0.25 ml 0.9% NaCl) were applied (75 μl i.v.), followed by constant infusion of both substances (2 mg/ml inulin, 5 mg/ml PAH) with a rate of 3.7 μl/h/300 g BW. The urinary bladder was catheterised to measure urine flow and to obtain urine samples. After reaching a steady state, urine was collected for 20 min and blood samples were drawn subsequently, centrifuged and supernatants were stored at −20°C. Inulin concentrations of the urine and the plasma were determined by fluorescence spectrometry (1420 Victor^2^Multilabel Counter, PerkinElmer, Turku, Finland), whereas PAH concentrations were measured by photospectrometry (Dynatec Labs, Guernsey, UK) using the anthrone method. Calculations of inulin clearance and PAH clearance were performed according to the following equations: Inulin clearance = (*I*_U_ × *V*_U_)/(*I*_P_ × *t*); PAH clearance = (PAH_U_ × *V*_U_)/(PAH_P_ × *t*); and PNS = [(PAH_U_ × *V*_U_)/*t*] − [GFR × PAH_P_] where *I*_U_ is inulin concentration in urine; PAH_U_ is PAH concentration in urine; *I*_P_ is inulin concentration in plasma; PAH_P_ is PAH concentration in plasma; *V*_U_ is urine volume; *t* is time of measurement and PNS is PAH net secretion.

### Markers of acute kidney injury and cytokine

At the end of the experiment, plasma samples were drawn to measure creatinine and urea by using routine laboratory methods. Cystatin C and NGAL were measured as previously described using rat NGAL (Kit 041 Dianova, Hamburg, Germany) and cystatin C (cystatin C ELISA Kit, AXXORA, Lörrach, Germany) kits [5]. The quantification of interleukin-6 (IL-6) was done with the Luminex-method (Invitrogen, Karlsruhe, Germany) as previously described [8].

### Histopathology

Tissues were harvested and stained with haematoxylin and eosin reagent and PAS reaction as previously described [5]. The morphological alterations of kidneys were analysed semi-quantitatively by a blinded investigator (12 random fields per view per section), where 0 was given when no alterations were found, 1 for mild alterations, 2 for medium alterations and 3 for severe alterations based on a previously established scoring system for AKI [5]. Criteria for histopathological assessment were formation of interstitial oedema, cellular oedema, detachment of tubular epithelium from the basement membrane, loss of the brush border of the proximal tubular cells, cell death and vacuolisation. Mean values from the scores of the latter criteria were taken together as total injury score.

### Statistical analysis

Values are expressed as mean ± SD or with boxplots. In boxplots, the central line marks the median and the height of the box represents the inter quartile range (IQR) with a confidence interval of 95%. Whiskers represent 1.5 × IQR. Statistical analyses were performed using SPSS V20. For parametric parameters, possible differences were assessed with ANOVA followed by *post hoc* Duncan test. Statistical significance is assumed for *p* < 0.05. For non-parametric data, Kruskal-Wallis following Mann-Whitney *U* tests with Bonferroni correction were used for significant differences.

## Results

### Induction of sepsis without mortality

**Figure 3:**
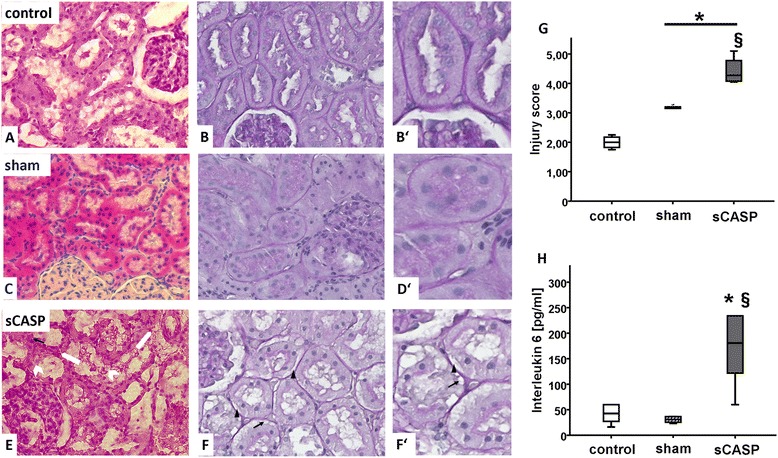
**Histology of AKI in a clinically relevant model of sCASP-induced sepsis.** HE staining of rat kidney cortex 24 h after sCASP surgery **(E)**, sham surgery **(C)** or control group **(A)**. Periodic acid Schiff (PAS) staining of rat kidney cortex 24 h after sCASP surgery **(F)**, sham surgery **(D)** or control group **(B)**. Some of the lesion criteria are exemplified. Therefore, magnifications of some areas are shown on the right (as indicated by the white big arrows). White arrows without heads in **(E)** point to interstitial edema. White arrowheads in **(E)** show tubular epithelial cells going to death. Black arrows in **(E)**, **(F)**, and **(F′)** show vesicles in epithelial cells of the tubules. Black arrowheads in **(F)** and **(F′)** show ablation of the tubular epithelium from the basement membrane. For details, see text. **(G)** shows the total injury score, whilst **(H)** provides evidence of systemic inflammation by showing different values of interleukin 6 [pg/ml]. *p < 0.05 vs. control, §*p* < 0.05 vs. sham.

First of all, we tested different sizes of intravenous cannulas (20, 18, 16, 14 G) as a colon stent. However, in these experiments the seminal vesicle of the rats occluded the stents and therefore only abscess cavities with local infection were formed. Because of the lack of sufficient systemic infection or peritonitis, we tested the tip of a special prepared suction catheter with a diameter of 10 F (Figure 2). Using this set up, all animals survived, revealed MAP > 70 mmHg during 24 h (with fluid resuscitation) and showed no abscess formation at the end of the experiments. sCASP-treated animals displayed clinical features of illness and showed ubiquitous peritonitis and significantly increased sepsis signs after 24 h including a decrease in activity, reduced alertness, ruffled fur, and hunched posture. This clinical status deteriorated continuously over time. Control and sham animals appeared outwardly normal. No significant differences in core temperature were detectable between groups. The fluid resuscitated sCASP group showed significant differences in MAP (80 ± 11 [mmHg]) and heart rate (446 ± 56 [beats/min]) and no differences in cardiac output (453 ± 72 [ml/kg/min] compared to control (92 ± 9; 379 ± 55; 378 ± 35) or sham (98 ± 17; 447 ± 112; 383 ± 48) (Table 1). The sCASP group showed no significant difference in RBF, when compared with sham (8.3 ± 1.2 [ml/min] vs. 9.1 ± 0.9 [ml/min], data not shown). Blood analyses after 24 h revealed signs of sepsis in sCASP-treated animals with increased lactate (3.0 ± 1.5 [mg/dl]; Table 2), increased IL-6 (251.4 ± 238 [pg/ml]; Figure 3) and a typical leucopenia for these animals (1.35 ± 0.59 [10^3^/μl]; Table 3) compared to control (1.7 ± 0.2; 41 ± 20; 3.00 ± 1.06) and sham (1.5 ± 0.3; 39 ± 23; 2.17 ± 1.34) (See Table 4).

**Table 1:** **Macrohaemodynamics**

	**HR** ^**a**^ **[beats/min]**	**MAP** ^**a**^ **[mmHg]**	**BR** ^**a**^ **[breaths/min]**	**HR** ^**b**^ **[beats/min]**	**MAP** ^**b**^ **[mmHg]**	**BR** ^**b**^ **[breaths/min]**	**CI** ^**b**^ **[ml/min/kg]**	**SVI** ^**b**^ **[ml/beat/min]**	**TPRI** ^**b**^ **[mmHg/ml/min/kg]**	**DO**_**2**_ **-I** ^**b**^ **[ml/min/kg]**
Control	382 ± 25§	115 ± 11	78 ± 12	378 ± 35	92 ± 9	99 ± 12	379 ± 55	1.11 ± 0.19	0.31 ± 0.15	70.4 ± 15.6
Sham	428 ± 22	120 ± 7	72 ± 10	383 ± 48	98 ± 17	92 ± 14	447 ± 112	1.24 ± 0.25	0.28 ± 0.11	76.5 ± 18.8
sCASP	388 ± 22§	128 ± 6	64 ± 10	446 ± 56*§	80 ± 11§	107 ± 23	453 ± 72	1.00 ± 0.20	0.26 ± 0.04	81.7 ± 16.3

^a^Baseline measurements (see Figure 1); ^b^intra-OP measurements (see Figure 1). HR, heart rate; MAP, mean arterial pressure; BR, breathing rate; CI, cardiac index; SVI, stroke volume index; TPRI, total peripheral resistance index; DO_2_-I, oxygen delivery index, *p < 0.05 vs. control, §*p <* 0.05 vs. sham.

**Table 2:** **Intra-OP BGA**

**BGA II**	**pH**	**pCO**_**2**_ **[kPa]**	**pO**_**2**_ **[kPa]**	**Hct [%]**	**Hb [g/dl]**	**sO**_**2**_ **[%]**	**Lactate [mmol/L]**	**HCO**_**3**_^**−**^ **[mmol/L]**	**SBE [mmol/L]**
Sham	7.40 ± 0.02	5.99 ± 0.30	18.78 ± 5.88	37.9 ± 2.9	12.4 ± 0.8	96.4 ± 1.1	1.5 ± 0.3	26.9 ± 1.5	3.0 ± 1.6
Control	7.45 ± 0.02	5.05 ± 0.41	24.69 ± 3.23	38.2 ± 3.3	12.4 ± 1.1	97.5 ± 0.3	1.7 ± 0.2	25.8 ± 1.2	2.1 ± 0.9
sCASP	7.41 ± 0.09	5.44 ± 1.06	23.97 ± 10.79	39.2 ± 9.0	12.8 ± 3.0	89.9 ± 17.8	3.1 ± 1.6§	25.1 ± 2.6	0.9 ± 3.2

§*p* < 0.05 vs. sham.

**Table 3:** **Common laboratory values**

	**Shame**	**Control**	**sCASP**
Sodium [mmol/L]	141 ± 3	142 ± 2	144 ± 3
Potassium [mmol/L]	5.2 ± 0.6	5.4 ± 0.2	5.0 ± 0.5
Calcium [mmol/L]	2.55 ± 0.06	2.42 ± 0.08	2.35 ± 0.21
Chloride [mmol/L]	107 ± 4	108 ± 2	112 ± 3*§
Osmolality [mosmol/kg]	299 ± 5	306 ± 3	315 ± 16
Glucose [mg/dl]	119 ± 21	121 ± 21	161 ± 50
ALAT [U/L]	83 ± 33	128 ± 42	355 ± 345*
ASAT [U/L]	33 ± 6	45 ± 10	141 ± 152*
GGT [U/L]	1.1 ± 1.4	1.1 ± 0.4	6.0 ± 8.1
Alkaline phosphatase [mmol/L]	90 ± 13	90 ± 10	157 ± 99
LDH [U/L]	646 ± 256	155 ± 90	803 ± 581*
Protein [g/dl]	3.9 ± 0.3	4.1 ± 0.3	3.8 ± 0.2
Albumin [g/dl]	2.2 ± 0.1	2.3 ± 0.2	2.1 ± 0.3
INR	0.81 ± 0.07	0.78 ± 0.03	0.79 ± 0.14
PTT [s]	20.6 ± 9.3	23.6 ± 10.5	24.2 ± 12.0
Leukocyte [Thsd/μl]	2.17 ± 1.34	3.00 ± 1.06	1.35 ± 0.59*
Erythrocyte [Mio/μl]	5.54 ± 0.67	5.84 ± 0.49	5.76 ± 0.84
Platelets [Thsd/μl]	200 ± 87	260 ± 94	139 ± 77

Values were determined after 27 h (see Figure 1); *p < 0.05 vs. control, §*p* < 0.05 vs. sham.

**Table 4:** **Baseline BGA**

**BGA I**	**pH**	**pCO**_**2**_ **[kPa]**	**pO**_**2**_ **[kPa]**	**Hct [%]**	**Hb [g/dl]**	**sO**_**2**_ **[%]**	**Lactate [mmol/L]**	**HCO**_**3**_^**−**^ **[mmol/L]**	**SBE [mmol/L]**
Control	7.45 ± 0.02	5.64 ± 0.44	12.19 ± 0.41	46.2 ± 1.6	15.1 ± 0.6	96.1 ± 0.4	1.1 ± 0.2	28.9 ± 1.6	5.0 ± 1.4
Sham	7.42 ± 0.02*	5.59 ± 0.26	11.71 ± 0.44	46.4 ± 1.3	15.3 ± 0.5	95.1 ± 0.9	1.1 ± 0.3	27.0 ± 1.9	2.8 ± 2.2
sCASP	7.37 ± 0.02*§	6.50 ± 0.66	13.00 ± 0.96§	48.9 ± 4.9	16.9 ± 1.7	95.2 ± 1.3	1.9 ± 0.6*§	27.5 ± 2.4	3.1 ± 2.0

*p < 0.05 vs. control, §*p* < 0.05 vs. sham.

**Table 5:** **Kidney function parameters**

**Kidney function parameters**	**Urine output [ml/20 min/100 g]**	**Cystatine C urine [pg/ml]**
Control	0.10 ± 0.05	1,249 ± 649
Sham	0.19 ± 0.14	1,011 ± 219
sCASP	0.05 ± 0.03§	1,846 ± 533

Values were determined after 27 h (see Figure 1). §*p* < 0.05 vs. sham.

### Determination of sepsis-induced acute kidney injury

#### Functional tests

**Figure 4:**
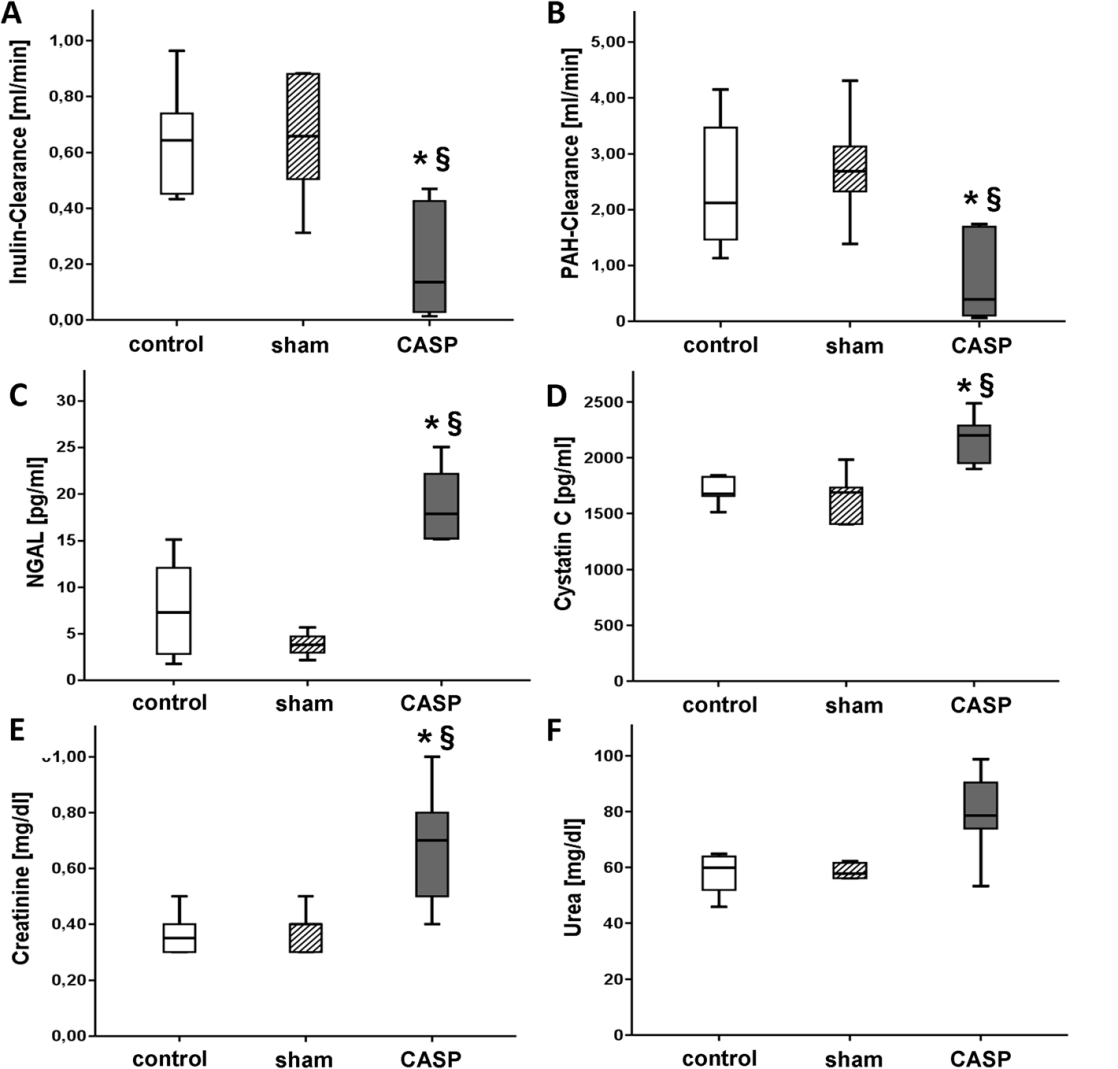
**Kidney function parameters.** Showing the gold standard of kidney function parameters inulin **(A)** and PAH clearances **(B)** [ml/min], as well as NGAL **(C)** and cystatin C **(D)** [pg/ml] in serum. Mentioning also the common clinical standard serum parameters creatinine **(E)** and urea **(F)** [mg/dl]. *p < 0.05 vs. control, §*p* < 0.05 vs. sham.

First, we measured the inulin clearance as the gold standard of the kidney function. After 24 h, the sCASP group had a significantly decreased inulin clearance of 0.20 ± 0.20 [ml/min] compared to sham (0.65 ± 0.23) and control (0.65 ± 0.20) (Figure 4A). In the same line, urine output decreased significantly ((0.05 ± 0.03 [ml/20 min/100 g]); Table 5). Additionally, PAH clearance was also significantly reduced to 0.80 ± 0.85 [ml/min] when compared to sham (2.76 ± 0.97) and control (2.47 ± 1.30) (Figure 4B).

#### Serum markers

The standard parameters for AKI such as urea [mg/dl] and creatinine [mg/dl] in serum changed from baseline levels in all animals (urea 42 ± 9 mg/dl and creatinine 0.43 ± 0.1 mg/dl; data not shown) to elevated levels in sCASP animals ((79 ± 17; 0.68 ± 0.21) Figure 3C,D), whereas only the creatinine level reached significance. Cystatin C [pg/ml] (2,171 ± 219) as well as NGAL [pg/ml] (19.1 ± 4.4) revealed significantly increased serum levels when compared to sham or control.

#### Histopathology

**Figure 5:**
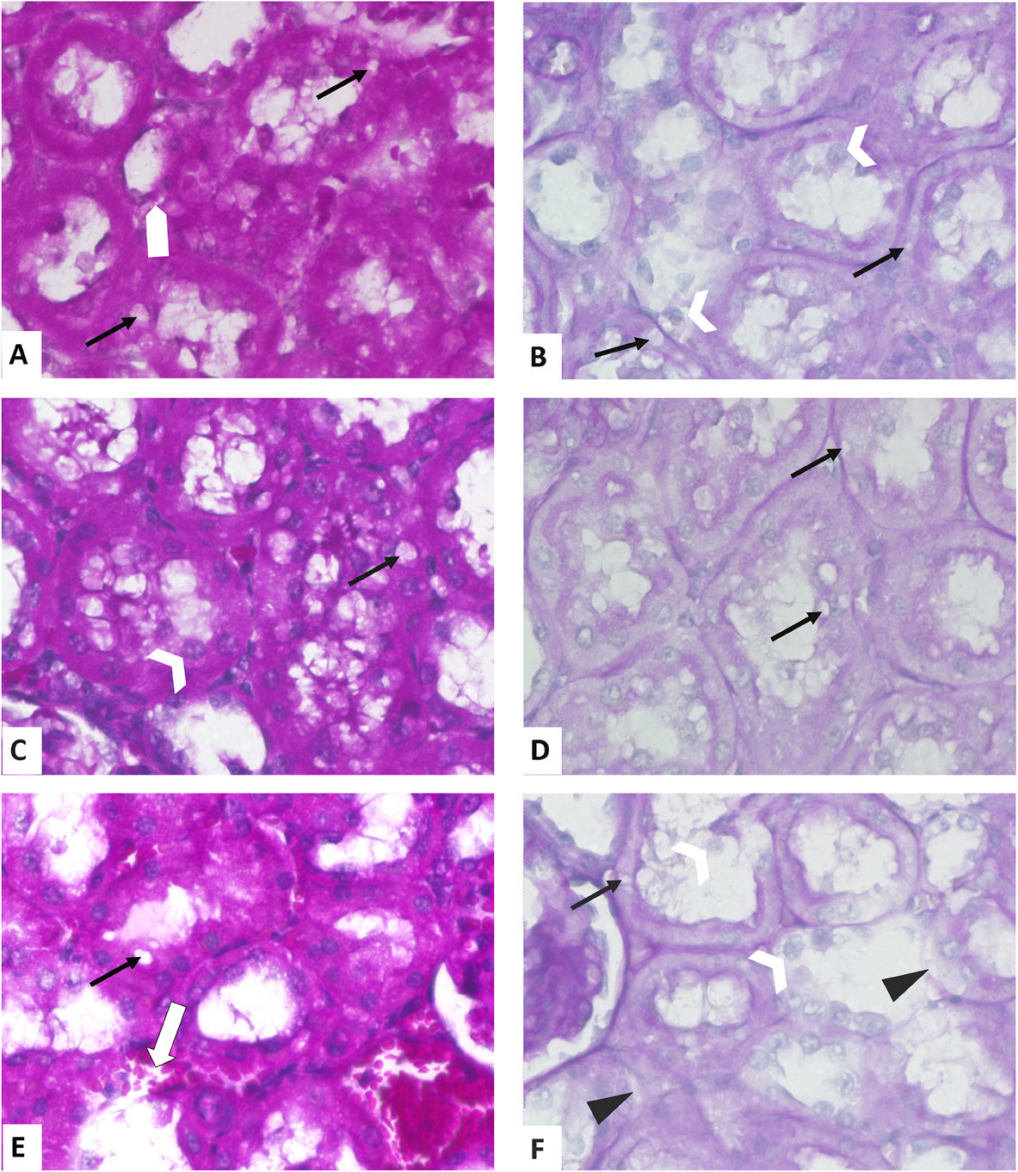
**HE and Periodic acid Schiff (PAS) staining of rat kidney cortex.** HE staining of rat kidney cortex 4 **(A)**, 6 **(C)** and 10 days **(E)** after sCASP surgery. Periodic acid Schiff (PAS) staining of rat kidney cortex 4 **(B)**, 6 **(D)** and 10 days **(F)** after sCASP surgery. White arrow without head in **(A)** points to interstitial edema. White arrowheads in **(B)**, **(C)**, and **(F)** show tubular epithelial cells going to death. Black arrows in **(A–F)** show vesicles in epithelial cells of the tubules. Black arrowheads in **(F)** show ablation of the tubular epithelium from the basement membrane. White arrow with black frame in **(E)** shows diffuse bleeding.

Interestingly, the sham procedure had a slight impact on the kidney, which can be seen in the total injury score (Figure 3 and 5). Furthermore, abdominal sepsis led to a significant increase of the histopathological injury score, when compared to control as well as to sham. In the kidneys of the sCASP group after 24 h, we could identify significantly more interstitial oedema when compared to sham or control, as well as an increased disappearance of the proximal tubules' brush border. The kidneys of the sCASP group showed significantly more dead cells when compared with sham and control. However, the leading reasons for the increased histopathological injury score in sCASP animals were the amount of interstitial oedema and the proximal tubules' brush border when compared with sham and control. Focusing on the detachment of the basement membrane, an insignificant increase could be detected in sCASP animals.

## Discussion

Septic AKI is caused by multifactorial impacts on the kidney. Hypovolemia and hypotension are not the only explanations for AKI in sepsis, because *in vitro* even serum from septic patients is harmful to human renal proximal epithelial tubular cells (PTC) [9]. The assumption that reduced renal blood flow (RBF) is causative for septic AKI is contrary to results that, even with increased RBF, septic AKI can occur in human as well as in animal investigations [10]. Renal microcirculation disorders and/or inflammatory stimuli by cytokines or bacterial fragments induce cell alarming cascades [3]. It can be hypothesized that compromised PTC in sepsis crosstalk in a paracrine way among themselves via, e.g. TNF-α [3]. The response to injury leads to metabolic downregulation and optimisation of cellular energy consumption in PTC. Thus, PTC sacrifice cellular function (e.g. detoxification) to limit further injury and to regain cellular metabolism faster after danger was abated. This orchestra of the response to sepsis leads to perilous reduction of renal function.

Only a few aspects of septic AKI are understood and clinical research in humans will not be able to give insights of ongoing histopathology changes, microcirculation disturbances or drug interactions on cellular levels. It has been postulated previously that there is a need for better animal models to investigate acute kidney injury associated with sepsis [11]. The CASP model, first published 1997 by Zantl et al. [12] was designed as an endogenous faecal contamination model. A stent of a defined diameter is implanted into the colon ascendens as an open connection between the intestinal lumen and the abdominal cavity [12]. The septic focus leads to the continuous spreading of bacteria, and this model allows the clearance of the sepsis focus by removal of the stent. It has been shown previously that depending on the size of the stent, the survival rate starts to decline after 24 h in rodent CASP models [13]. Injection of LPS, which is more likely a hyperinflammation storm rather than sepsis, and the CLP method are both the most commonly used animal models for septic AKI research. The CLP model served as the gold standard sepsis model, however with numerous limitations [14].

The amount of stool in the coecum varies widely and cannot be predicted, even when animals are fasted before CLP. Thus, the level of sepsis due to caecum perforation and faeces efflux changes between animals during the same trial. Another unpredictable variation of the CLP procedure is the possible spontaneous closure of the perforation (by the gut itself or, e.g. by the omentum). Furthermore, the CLP procedure starts with the ligation of the caecum, representing an ischemic hit and no initial septic insult. Taken together, AKI in CLP models is the result of an ischemic coecum and a faecal peritonitis of an unpredictable degree [15]. Another promising approach used the intraperitoneal injection of faecal slurry to induce a peritoneal sepsis in rodents, resulting after 6 h in decreased oxygen delivery in various organs and after 24 h in global organ dysfunction [16]. This model results in a substantial septic insult; however, this model was not designed to mimic and resemble the pathophysiology found in human postoperative abdominal sepsis.

Therefore, we established a new animal arrangement for septic AKI induced by colon stent peritonitis. We used male rats to exclude hormone variation during investigations. Because omentum and seminal vesicles occluded the stents during the first experiments, we enlarged the size of the stent to 10 F and modified the stent for the induction of a homogeneous sepsis. Using this set up, we were able to induce sepsis within 24 h followed by AKI without increase in mortality. Consequently, the presented model allows studying AKI induced by a defined faecal peritonitis, without unpredictable loss of animals due to multiple organ failure [15,13]. We are able to show an increased kidney injury in kidney specimens from animals 4, 6 or 10 days after the sCASP procedure (Figure 5). The virtue of our setup is to study even the delayed onset of AKI (after several days), simulating the human clinical setting, because in human sepsis, AKI occurrence reaches a plateau after numerous days [17]. Many published trials regarding AKI did not measure macrohaemodynamics and therefore ignored pre-renal failure caused by hypotension. Septic animals often show respiratory failure with decreased PaO_2_ [16]. Thus, tissue hypoxia can influence renal integrity and may affect morbidity and mortality [18]. Therefore, we established an intensive care set up involving fluid resuscitation (14 ml/kg BW/24 h crystalloids including i.v. medication), continuous observation of macrohemodynamics as well as sufficient oxygenation (using blood gas analyses). It has been shown previously that the opioid fentanyl influences the immunity after surgery [19]. However, the necessity of sufficient analgesia in this model and the fact that fentanyl is one of the most used opioids in clinical ICU settings led us to use fentanyl in all our animals to exclude any confounding effect between groups.

In the present study, AKI secondary to sepsis was documented by functional and morphological data. RBF was not different between sCASP and sham, showing that AKI in our model was not due to global renal hypoperfusion. As shown previously, stable macrohaemodynamics in our model remained near the lower limit of the renal auto-regulatory range reported for anaesthetized rats [20].

Routine serum parameters of kidney injury (creatinine and urea) were elevated, as well as serum NGAL, a PTC damage marker [21], and serum cystatin C, an early predictor for AKI [22]. However, it has been shown recently that NGAL and cystatin C (in rodent urine and serum) exhibit low sensitivity and specificity for glomerular and tubular injury [23]. Thus, inulin and PAH clearance, the gold standard for renal function, were used to determine kidney function. sCASP showed a decreased inulin clearance, whilst PAH clearance and urine output were also reduced when compared to sham and control. Corrigan et al. stated since substantial time that PAH clearance does not reflect renal perfusion [24]. PAH clearance is a valid measure for renal blood flow only if renal extraction of PAH is almost complete and if renal extraction is not changed due to experimental conditions. It has been shown recently that the reduced function of the tubular rate limiting basolateral organic anion transporters (OAT1 and OAT3) influenced PAH clearance [25]. Since RBF in sCASP animals in our study was not different to control animals, PAH clearance reflects PAH extraction and kidney function.

Conflicting results were published about the importance of histopathology for septic AKI investigation. In our opinion, histopathology plays an important part in the AKI research, because compromised histology, e.g. loss of brush border, are detectable even when serum parameters for renal failure are still showing standard values. Furthermore, it is possible to detect specific characteristic deposition patterns of drugs, as previously published [5]. Therefore, tissue examinations give essential information for follow-up investigations.

The animals used in our survey have been 8 to 12 weeks old. It is well known (in humans and animals) that the severity of sepsis-induced acute kidney injury is age dependent [26,27]. However, the purpose of our work was to establish a stable rodent set up of septic AKI being close to the clinical ICU setting. Consequently, further ongoing studies focus on sCASP-induced AKI in aged animals.

## Conclusions

Taken together, the presented sCASP method, leading to septic AKI, is a standardized, reliable and reproducible method in comparison to CLP procedure. Stable development of septic AKI, monitoring and stabilisation of macrohemodynamics and gas exchange, low mortality rate and the opportunity to observe kidney function with clinically relevant methods are the advantages of this new model. Thus, our described method may serve as a new standard for experimental investigations of septic AKI.

## Abbreviations

AKI: acute kidney injury
BW: body weight
CO: cardiac output
FiO_2_: fraction of inspired oxygen
IL-6: interleukin-6
LPS: lipopolysaccharides
MAP: mean arterial pressure
NGAL: neutrophil gelatinase-associated lipocalin
PAH: para-aminohippuric acid
PAS: periodic acid-Schiff
PaCO_2_: partial pressure of carbon dioxide
PaO_2_: partial pressure of oxygen
PTC: proximal epithelial tubular cells
RBF: renal blood flow
SaO_2_: oxygen saturation
sCASP: standardized colon ascendens stent peritonitis
